# High-Speed Imaging of Giant Unilamellar Vesicle Formation in cDICE

**DOI:** 10.1021/acsomega.4c04825

**Published:** 2024-09-25

**Authors:** Lori Van de Cauter, Yash K. Jawale, Daniel Tam, Lucia Baldauf, Lennard van Buren, Gijsje H. Koenderink, Marileen Dogterom, Kristina A. Ganzinger

**Affiliations:** † Autonomous Matter Department, AMOLF, Amsterdam 1098 XG, The Netherlands; ‡ Department of Bionanoscience, Kavli Institute of Nanoscience, Delft University of Technology, Delft 2629 HZ, The Netherlands; § Laboratory for Aero and Hydrodynamics, Delft University of Technology, Delft 2629 HZ, The Netherlands

## Abstract

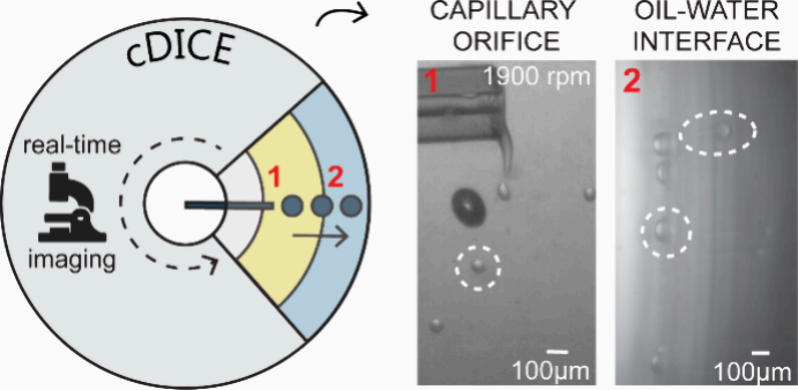

Giant unilamellar vesicles (GUVs) are widely used as in vitro model membranes in biophysics and as cell-sized containers in synthetic biology. Despite their ubiquitous use, there is no one-size-fits-all method for their production. Numerous methods have been developed to meet the demanding requirements of reproducibility, reliability, and high yield while simultaneously achieving robust encapsulation. Emulsion-based methods are often praised for their apparent simplicity and good yields; hence, methods like continuous droplet interface crossing encapsulation (cDICE), which make use of this principle, have gained popularity. However, the underlying physical principles governing the formation of GUVs in cDICE and related methods remain poorly understood. To this end, we have developed a high-speed microscopy setup that allows us to visualize GUV formation in real time. Our experiments reveal a complex droplet formation process occurring at the capillary orifice, generating >30 μm-sized droplets and only in some cases GUV-sized (∼15 μm) satellite droplets. According to existing theoretical models, the oil–water interface should allow for the crossing of all droplets, but based on our observations and scaling arguments on the fluid dynamics within the system, we find a size-selective crossing of GUV-sized droplets only. The origin of these droplets remains partly unclear; we hypothesize that some small GUVs might be formed from large droplets sitting at the second interface. Finally, we demonstrate that proteins in the inner solution affect GUV formation by increasing the viscosity and altering the lipid adsorption kinetics. These results will not only contribute to a better understanding of GUV formation processes in cDICE but ultimately also aid in the development of more reliable and efficient methods for GUV production.

## Introduction

The quest to understand and manipulate the building blocks of life, including the countless interacting molecules and biochemical reactions making up cellular life, is a major aim of biophysics and synthetic biology.^1^ One key tool in these fields is giant unilamellar vesicles (GUVs) as cell-sized, lipid bilayer-enclosed reaction compartments.^2,3^ Since their first description^4^ in 1969, GUVs have proven to be a powerful and versatile tool as they can be directly observed using real-time microscopy and easily manipulated using biophysical tools, making them ideal in vitro model membrane systems.^3,5,6^ More recently, GUVs have also been proposed as containers for a future synthetic cell^7−10^ and as reaction containers for chemistry and more complex cargo carriers in drug delivery.^11,12^

Despite the widespread research use of GUVs, there is still no one-size-fits-all method for their production.^10^ Over the years, numerous methods have been developed to meet the demanding requirements of reproducibility, reliability, and high yield while simultaneously achieving robust encapsulation. Historically, swelling-based methods (natural swelling,^4^ electroformation,^13−16^ and gel-assisted swelling^17−20^) have been used extensively for studying the biophysical properties of membranes. However, these easy-to-implement, high-yield methods offer poor control over the encapsulation efficiency and the stoichiometry of encapsulated molecules. Thus, they offer only limited compatibility with establishing complex reconstituted systems. Emulsion-based techniques (w/o droplets crossing an oil–water interface using gravity, centrifugation, microfluidic devices, or microfluidic jetting^21−27^), on the other hand, offer more control over GUV content and enable experiments with complex encapsulated contents. Despite the potential cost of residual membrane impurities,^10,28,29^ emulsion-based methods have therefore gained popularity in recent years.

One method that particularly gained a lot of traction is called continuous droplet interface crossing encapsulation (cDICE).^30−36^ In cDICE, water-in-oil (w/o) droplets that are produced at a capillary orifice are continuously forced through an oil–water interface by centrifugal force in a rotating chamber, thereby forming a lipid bilayer and thus GUVs.^30^ Recent optimization has made the method compatible with a wide range of biological systems, thereby offering control over encapsulated content, a high GUV yield, and straightforward implementation.^31^ However, our understanding of up to which degree the encapsulated contents’ complexity in cDICE can be extended, with respect to both physical properties (e.g., viscosity of encapsulated fluid) and physicochemical properties (e.g., which proteins and protein systems), remains limited. While many successes have been celebrated using cDICE, we still do not understand the underlying GUV formation process and how this affects the inherent variability in content encapsulation and yield seen in cDICE.^10^

To gain a deeper understanding of GUV formation in cDICE, we have developed a high-speed microscopy setup that allows us to visualize the GUV formation process inside the rotating chamber in real time. We focused on the capillary orifice, where initial droplet formation occurs, and on the oil–water interface, where droplets are converted into GUVs. Our experiments reveal a complex droplet formation process occurring at the capillary orifice, governing the formation of both larger droplets and, likely, satellite droplets of the size of typical cDICE GUVs (12 μm being the average diameter of GUVs formed with cDICE^31^) in some cases. The transfer of these droplets through the oil–water interface appears to exhibit selectivity toward GUV-sized droplets that may also be formed from large droplets at the second interface. We support these experimental observations with scaling arguments. Finally, we demonstrate that the addition of a protein to the inner solution increases the viscosity and alters the kinetics of lipid adsorption, thereby significantly influencing the process of GUV formation.

## Results and Discussion

### Design of an Imaging Setup to Visualize Droplet and GUV Formation in cDICE

In the cDICE method, the initial step of GUV formation is the generation of droplets at a capillary orifice, which is inserted perpendicularly into the oil layer in the rotating chamber. In its original implementation, cDICE uses a capillary diameter of 2–25 μm to allow for tight control over GUV sizes.^30^ However, we and others found such narrow capillaries to be very impractical when encapsulating protein solutions as these capillaries are prone to rapid clogging, leading to highly irreproducible results. In our previous work, we showed that this issue can be circumvented by using wide capillaries with a diameter of 100 μm.^31^ The flow regime is therefore significantly different from the original protocol,^30^ and one would not necessarily expect tight control over droplet sizes. Still, we found that these capillaries produced a surprisingly narrow size distribution of GUV sizes, roughly ten times smaller than the capillary orifice (∼10 vs ∼100 μm).^31^

**Figure 1:**
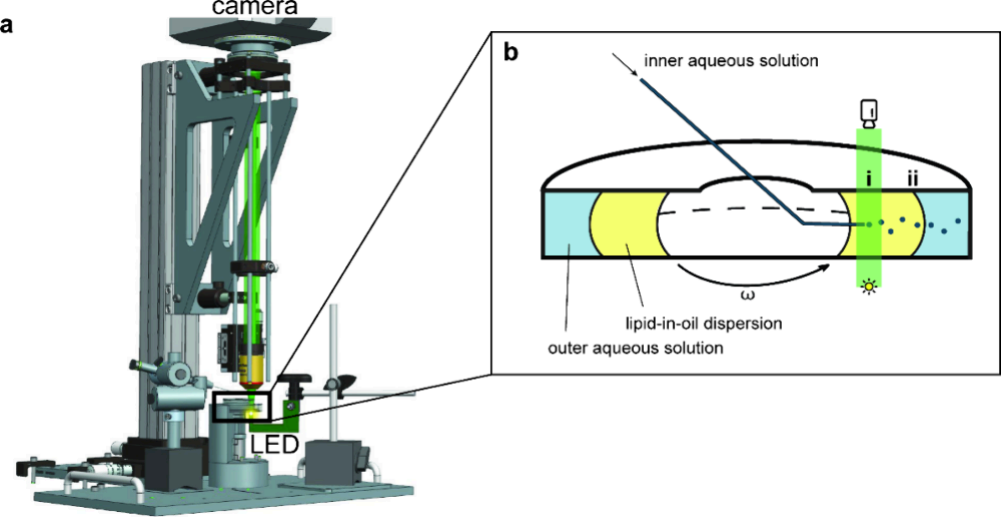
Development of a high-speed imaging setup to visualize GUV formation in cDICE. (a) The imaging setup consists of a high-speed camera suspended above the rotating chamber and an intense light source located directly below the rotating chamber. For an interactive 360° view of the setup, see the Methods section. (b) In cDICE, (i) aqueous droplets are generated at the capillary orifice; subsequently, they travel outward through the lipid-in-oil dispersion (LOD); and finally (ii) traverse the oil–water interface, where droplets are converted into GUVs.

To better understand how a large capillary orifice can still lead to such a relatively monodispersed GUV size distribution in cDICE, we developed a high-speed microscopy setup to, for the first time, visualize the processes of droplet and GUV formation in cDICE in real time (Figure 1). We designed the setup so that the camera is suspended vertically above the cDICE apparatus, capturing the light of a light source located directly beneath the rotating chamber (see the Methods section for a full description of the setup; Figure 1a). This way, we are able to capture the process along the horizontal axis of the rotational chamber: from the capillary orifice, where initial droplet formation occurs (Figure 1b (i)), to the oil–water interface, where droplets are converted into GUVs (Figure 1b (ii)). Due to the high rotation speeds that are used in cDICE (∼1900 rpm), all processes happen on a very fast time scale, on the order of microseconds (10^–6^–10^–5^ s). To obtain a sufficiently high time resolution, we therefore used fast cameras in combination with brief exposure times up to 1 μs, reaching frame rates up to 30,000 fps.

### Droplet Formation at the Capillary Orifice Is Governed by Shear Forces

**Figure 2:**
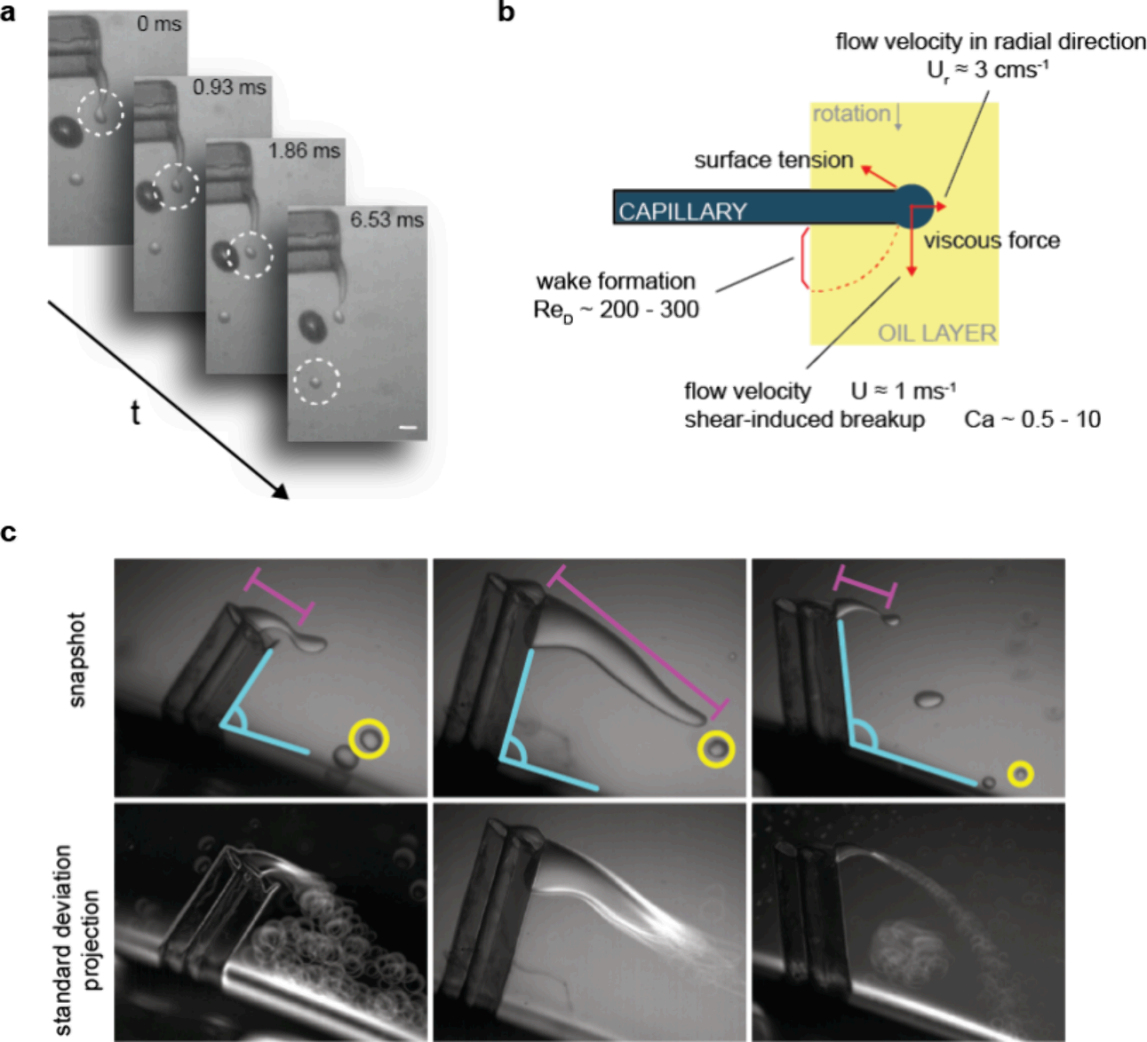
Droplet formation at the capillary orifice is governed by shear forces. (a) Microscopic image sequence capturing a droplet of PBS buffer with 18.5% v/v OptiPrep being sheared off from the liquid stream at the capillary orifice at a rotation speed of 1900 rpm. Scale bar is 100 μm. (b) Illustration depicting the different forces acting at the capillary orifice: the *Ca* capillary number (*Ca* ∼ 0.5–10) indicates a shear-induced breakup mechanism, while the Reynolds number (*Re_D_* ∼ 200–300) describes the wake formation behind the capillary. The shear velocity (*U* ≈ 1 ms^–1^) is larger than the flow velocity in the radial direction (*U*_*r*_ ≈ 3 cms^–1^), further indicating that droplet formation is shear-induced. (c) Microscopy images highlighting the high interexperiment variability using the same capillary in consecutive independent experiments. For identical experimental conditions, noticeable differences can be seen, e.g., in the insertion angle (top row, cyan), liquid filament (top row, magenta), and droplet size (top row, yellow). Bottom row: standard deviation stack projection of 100 frames (every 50th frame of 5000 frames). White highlights indicate variations in movement such as the occurrence of a droplet vortex in the wake of the capillary (left and right images) and the movements of the liquid filament end (left and middle).

When we focused our imaging setup on the capillary orifice at our default conditions for GUV production (100 μm diameter fused silica capillary, a rotation speed of 1900 rpm, and a flow rate through the capillary of 25 μL min^–1^; see the Methods section for further details), it immediately became clear that droplet formation under these conditions is a nonuniform, highly dynamic process with an irregular breakup pattern of a liquid filament into individual droplets (Figure 2a, Movie 1). Instead of the distinct droplet formation expected for low Reynolds numbers,^30,37^ we observed fluid exiting the capillary forming a liquid filament, which often adhered to the capillary. Droplet breakup took place at the end of the liquid filament at a fast rate, with droplet sizes clearly larger than the average cDICE GUV ((68.6 ± 2.8) μm, approximately 2500 droplets per second).

Upon silanization of the capillary, we no longer observed the fluid adhering to the capillary, resulting in a more regular droplet breakup mechanism (Figure S1). This can likely be explained by an increased surface hydrophobicity upon silanization when compared to the default polyimide capillary coating surrounding the capillary and the uncoated cut tip cross section with chipped coating edges, which results in less wetting of the capillary surfaces.

We observed significant variability in the droplet breakup dynamics at the end of the liquid filament. Factors contributing to this variability include irregularities in the capillary orifices resulting from suboptimal cutting or capillary deterioration over sustained use, differences in capillary insertion angle, and the occasionally observed presence of an air pocket at the base of the capillary (Figure 2a,c). Note that in all of these cases, the experimental condition was indistinguishable by eye, and the differences only became apparent when visualizing droplet formation with our dedicated imaging setup.

To better understand the observed droplet breakup mechanisms, we turned to scaling arguments to rationalize our findings (Figure 2b). Our video recordings (Movie 1) suggest that droplet breakup at the tip of the capillary is not due to inertial jetting but instead is induced by viscous shear stresses. For droplets forming from a capillary of diameter *D*, inertial jetting is expected for flow rates larger than a critical flow rate scaling with ∼ π(*D*^3^γ/2ρ_i_)^1/2^, where ρ_*i*_ ≳ 1 g/mL denotes the density of the inner solution (e.g., 1.0183 g/mL for the MRB80 buffer with 1.75% w/v sucrose) and γ is the interfacial tension between the dispersed and the continuous phases.^37^ In our experiments, the flow rate through the capillary is 25 μL min^–1^, which is significantly lower than the critical flow rate. This is consistent with our observation that droplets are indeed sheared off of the capillary. Here, we must therefore consider the balance between surface tension and viscous forces characterized by the capillary number *Ca*. *Ca* is given by *Ca* = μ*U*/γ. The flow velocity *U* at the point of insertion of the capillary is *U* = Ω*R*_*i*_, where *R*_*i*_ is the distance between the capillary orifice and the center of rotation of the chamber and Ω is the rotation speed. With *R*_*i*_ ∼ 1 cm, Ω ∼ 1000–2700 rpm, μ ∼ 4–5 × 10^–3^ kg m^–1^s^–1^, and assuming an interfacial tension between the inner solution and the oil phase of γ ∼ 10^–3^–10^–2^ mN m^–1^,^31^ the capillary number ranges between 0.5 and 10. Monodispersed droplets form at the tip of the capillary through a dripping mechanism for low values of the capillary number.^30^ Within the higher range of *Ca* reached in our experiments, droplets are therefore expected to deform and the breakup mechanism to be unstable, in agreement with our observations.

cDICE experiments require high rotational speed (Ω > 1000 rpm), producing flow instabilities in the wake of the static capillary inserted into the rotation chamber. Indeed, the Reynolds number, characteristic of the flow around the capillary, *Re*_*D*_ = ρ*UD*/μ, yields values in the range of *Re*_*D*_ ∼ 200–300, with ρ ∼ 0.934 g/mL being the density of the LOD. For *Re*_*D*_ ≥ 47, periodic vortex shedding in the wake of a cylinder is expected,^38^ and for *Re*_*D*_ ≥ 150, further three-dimensional instabilities are predicted,^38^ suggesting that the wake around the capillary will also affect droplet breakup. Indeed, we observe oscillations in droplet breakup, caused by the nonlinear effects in the wake, and the inner solution adhering to the outer capillary surface. Additionally, we observed that the droplets did not immediately travel outward as expected but rather initially exhibited an inward movement in the wake of the capillary and toward the center of the rotating chamber, before traveling outward. The larger diameter of the capillary leads to a larger capillary number *Ca* and to a wake instability, both of which contribute to a less stable droplet breakup and a larger variation in droplet size compared to a previous work.^30^

### Droplet Size, in Contrast to GUV Size, Is Dependent on the Rotation Speed

**Figure 3:**
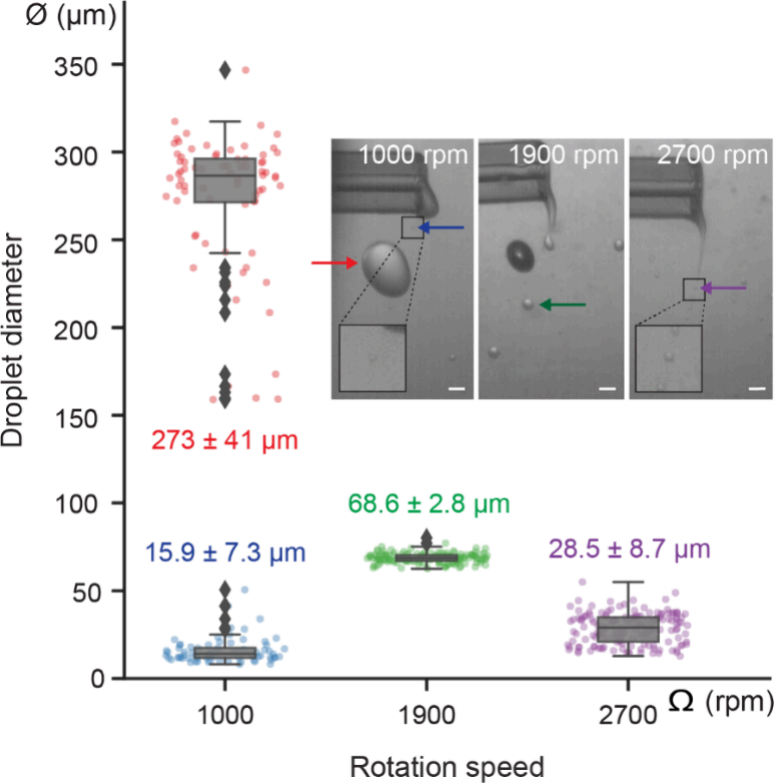
Size distributions of droplets for different rotation speeds (single column). Boxplots of droplet diameter φ at rotation speeds Ω of 1000, 1900, and 2700 rpm (*n* = 148, 152, and 157, respectively, for *N* = 1). Individual data points indicate single droplets and boxplots indicate medians and quartiles, while outliers are marked with individual diamond shapes. A rotation speed of 1000 rpm resulted in two distinct droplet populations: large droplets of mean diameter φ (273 ± 41) μm (red) and satellite droplets of mean diameter φ (15.9 ± 7.3) μm (blue). A rotation speed of 1900 rpm resulted in the narrowest distribution, with a mean droplet diameter φ of (68.6 ± 2.8) μm (green). 2700 rpm resulted in the smallest droplet sizes, with a mean diameter φ of (28.5 ± 8.7) μm (purple). Inset: Representative field-of-views for the different rotation speeds indicating the formed droplets with arrows. Scale bars indicate 100 μm.

To explore factors that influence droplet breakup in cDICE, we next altered the rotational speed of the rotating chamber. As the rotation speed of the chamber increases, the flow velocity at the capillary orifice also increases, and the viscous forces become stronger. This leads to the droplets being more likely to break up, resulting in smaller droplets. In line with this expectation, an increase in rotation speed to 2700 rpm resulted in smaller droplets formed at a higher frequency ((28.5 ± 8.7) μm and ∼34,500 droplets per second; Figure 3, Movie 2). Decreasing the rotation speed to 1000 rpm, the lowest speed at which oil and water layers maintain a vertical interface and GUVs can be produced, had the opposite effect, i.e., larger droplets formed at a lower frequency ((273 ± 41) μm and ∼40 droplets per second; Figure 3, Movie 3). We can estimate the droplet size from a force balance between the surface tension force ∼π*D*γ and the viscous force ∼6πμ*aU*, where *D* is the outer diameter of the capillary and *a* is the radius of the droplet.^37^ The droplet size above which breakup is expected scales with the inverse of the capillary number *a*/*D* ∼ (6 *Ca*)^−1^, and we predict a droplet diameter of ∼100 μm at 1900 rpm increasing to ∼200 μm when the rotation rate is decreased to 1000 rpm. These scalings are consistent with the order of magnitude of our experimental measurements (Figure 3). Droplet formation is thus shear-induced in a broad range of rotation speeds, encompassing both lower and higher speeds than the default of 1900 rpm. Our observation that droplet size is dependent on chamber rotation speed contrasts with the size distributions for GUVs obtained using these conditions: these distributions are all indistinguishable from one another and centered around 12 μm (Figure S2) and thus 3–30-fold smaller in diameter than the produced droplets. Hence, a large number of the droplets formed at the capillary are not directly converted into GUVs.

While a rotation speed Ω of 1900 rpm resulted in the narrowest droplet size distribution of all explored rotation speeds, interestingly, a rotation speed of 1000 rpm resulted in two distinct populations (Figure 3): one primary population of droplets with a mean diameter φ of (273 ± 41) μm and a secondary population consisting of smaller droplets with a mean diameter φ of (15.9 ± 7.3) μm. Occasionally, the formation of large and small droplets was disrupted when, e.g., a droplet merged with the liquid stream or collided with the capillary. Inspecting the videos more closely, we found that the observed population of small droplets consists of satellite droplets, produced when a bigger droplet breaks off from the main liquid thread at the tip of the capillary (Movie 3). Such satellite droplets have previously been observed in many breakup configurations, from T-junctions to the breakup of droplets in pure shear.^39^ While we did not observe any satellite formation for rotation speeds >1000 rpm, this may be due to our limited optical and temporal resolution: the satellite droplets observed for 1000 rpm (diameter ∼15 μm) were at the limits of our image resolution; droplets of any smaller diameter were too small to be identified and measured with sufficient certainty (see the Methods section for further details). It is therefore possible that satellite droplets of all sizes, within the size range of the final GUVs (1–20 μm), are also formed but not detected by our imaging setup.

In addition to the small satellite droplets that we observed at 1000 rpm, smaller droplets could theoretically also be formed when larger droplets break up due to shear forces generated in the flow by the rapid relative motion of the bottom wall of the rotational chamber with respect to the capillary. Droplets formed at the tip of the capillary are entrained by the flow in the rotation direction at a high velocity of *U* ≈ Ω*R*_*i*_ ≈ 1 m s^–1^ compared to the slow radial motion *U*_*r*_ = (ρ_*i*_–ρ_o_)*a*^2^Ω^2^*R*_*i*_/μ ≈3 cm s^–1^, determined by the balance between centrifugal and viscous forces. These droplets therefore interact with the wake left behind the capillary for several rotations. In the wake, the characteristic shear rate ε̇ scales with ε̇ ∼ Ω*R*_*i*_/*l*, where the characteristic length scale *l* for shear around the capillary will range between the outer diameter of the glass capillary ≈0.5 mm and the distance between the capillary and the bottom of the flow chamber ≈5 mm. One can define another capillary number as *Ca*_ε̇_ = με*ȧ*/γ, where *a* is the radius of the droplet.^40,41^ This number characterizes the relative magnitude of the viscous shear forces due to the shear rate ε̇ and the surface tension forces. *Ca*_ε̇_ = 1 corresponds to a condition where the smallest droplets cannot be further broken up by the shear^40,41^ and yields *a* ∼ γ/μ*ε̇*. Knowing *h* ∼ 0.5 cm, we find that the interfacial tension of the monolayer at the inner solution/oil interface needs to be approximately γ ∼ 10^–5^–10^–6^ N m^–1^ to produce droplets of *a* ∼ 5 μm, equivalent to the final GUV size. This value for an interfacial tension at an aqueous/oil interface is extremely low and not expected, even in the presence of surfactants or lipids. For reference, the interfacial surface tension between two miscible liquids is of the order 10^–6^ N m^–1^.^42^ Hence, we conclude that it is unlikely that GUV-sized droplets form by shear-force-induced droplet breakup after droplet formation at the capillary orifice.

### Protein in the Inner Solution Affects Viscosity and Lipid Adsorption

Next, we set out to study the effect of proteins on droplet formation in the capillary orifice. It is well-known that encapsulation of more complex solute mixtures, such as proteins and their associated buffers, leads to a decreased yield and variable encapsulation efficiencies.^31,43^ For cDICE specifically, it has been reported that the yield decreased at a high protein concentration,^44^ yet it is still unknown why this is the case. We also noticed both protein- and buffer-dependent effects on yield and encapsulation efficiencies, with MRB80 buffer and tubulin both resulting in worse outcomes than G-buffer and actin (Figure S3). To better understand what may be causing this difference, we chose to investigate the effect of these proteins on droplet formation, which was additionally motivated by the widespread efforts for cytoskeletal reconstitution inside GUVs.

**Figure 4:**
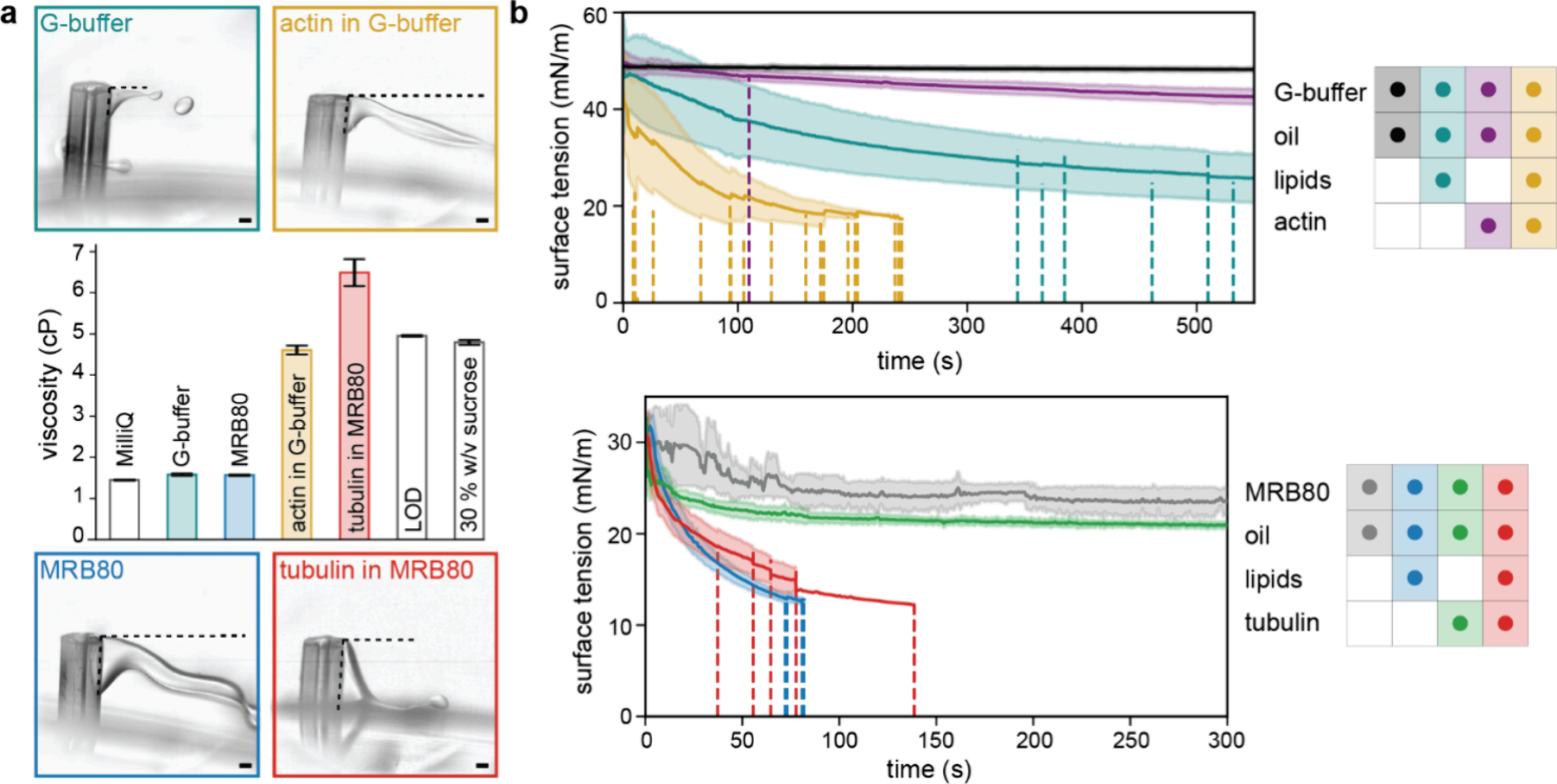
Effect of protein on aqueous solution properties. (a) Representative field-of-views of droplet formation at the capillary orifice for different buffer and protein solutions (actin 4 μM and tubulin 40 μM). Horizontal dotted lines indicate the liquid filament length just before the drop breaks off, while vertical dotted lines along the capillary indicate the extent of the external capillary surface wetted by the aqueous solution. Images are background-subtracted for better contrast. Scale bars indicate 100 μm. Middle: Dynamic viscosity measured using a parallel-plate rheometer for different buffers (G-buffer with 6.5% v/v OptiPrep, MRB80 with 1.75% w/v sucrose) and protein solutions (actin 1 μM and tubulin 33.33 μM), along with water (Milli-Q), LOD, and 30% w/v sucrose solution in MRB80 for reference. Error bars represent the standard deviation. (b) Interfacial tension kinetics measured using pendant drop tensiometry for different combinations of aqueous and oil solutions; G-buffer and actin 4 μM (top), and MRB80 and tubulin 33.33 μM (bottom). Solid lines represent the average values, and the shaded region corresponds to standard deviation. The vertical dotted lines represent the event of falling of a drop and truncation of the data.

Upon addition of either protein, droplet breakup at the capillary orifice also occurred at the tip of the liquid stream exiting the capillary. However, the oscillations of the liquid stream in the wake of the capillary were significantly reduced (Figure 4a, Movies 4, 5, 6, 7). Remarkably, in the case of tubulin, the liquid stream displayed a tendency to adhere to the air–oil interface. To explain these observations, we characterized the inner solution. We looked into both the physical properties, i.e., dynamic viscosity as determined by bulk shear rheology, and physicochemical properties, specifically the lipid adsorption rate determined from pendant drop tensiometry.

In the presence of actin and tubulin, the dynamic viscosity increased with respect to its accompanying buffer, G-buffer and MRB80 buffer, respectively (Figure 4a). For actin (1 μM in G-buffer, 6.5% v/v OptiPrep), an almost 3-fold increase from 1.58 to 4.61 cP was observed (Figure 4a, yellow bar), while for tubulin (33.33 μM in MRB80 buffer, 1.75% w/v sucrose), the viscosity increased 4-fold from 1.57 to 6.49 cP (Figure 4a, red bar). All solutions still exhibited a Newtonian fluid behavior. Important to note is that the used concentrations of added proteins remained within the micromolar range and are widely used in the field. Interestingly, the viscosity of the inner solution containing the protein was similar to the viscosity of the continuous phase, i.e., the surrounding LOD (Figure 4a, middle “LOD” bar). The fragmentation of the liquid filament into droplets at the end of the capillary is a consequence of complex instabilities beyond the scope of this study. These mechanisms are significantly affected by the viscosity of the inner solution, and the increased viscosity due to the added protein will dampen the flow dynamics in the liquid filament. This dissipation in the liquid stream can explain the decrease in the fluctuations observed in the liquid filament exiting the capillary (Figure 4a, Movies 4, 5, 6, 7). Moreover, previous studies on capillary breakup have reported that viscosity affects the fragmentation pattern and the size distribution of satellite droplets significantly. In particular, the viscosity increase in a liquid filament has been associated with fewer and larger satellite droplets.^45,46^ Therefore, proteins included in the inner solution can have a significant impact on the size distribution of the droplet formed at the capillary exit. Altogether, these results show a nuanced interplay between the physical properties of the encapsulation solution, varying with its composition even at low protein concentrations, and the fluid dynamic processes that govern droplet breakup.

To investigate how the addition of protein to the inner solution alters the physicochemical properties of the interface, we used pendant drop tensiometry^47^ to study lipid monolayer formation in a controlled environment. We analyzed the lipid adsorption kinetics and interfacial tension dynamics of the water–oil interface for different encapsulation solutions, mimicking droplet formation at the capillary orifice. It has been shown that proteins spontaneously adsorb at the oil–water interface and their behavior cannot unequivocally be attributed to a single protein property, with thermodynamic stability, structural properties, and concentration all being contributing factors.^48,49^ Particularly, actin has been shown to exhibit surface activity in a charge-dependent manner, influenced by both lipid and buffer composition, with a more pronounced effect observed for the filamentous form compared to actin monomers.^50−52^ Tubulin (specially β-tubulin inserts the amphipathic polymerizing interface into the DOPE membrane) is also shown to interact with the lipid membranes.^53,54^

Upon addition of 4 μM actin to the inner solution, a pronounced decline in interfacial tension was observed (Figure 4b, purple curve), with some droplets detaching before the end of the experiment (Figure 4b, dashed lines). This trend was consistent for tubulin (Figure 4b, green curve). To examine the roles of actin and tubulin as surface-active agents in the interfacial tension, we then compared the interfacial tension dynamics against those of a lipid-free oil dispersion. Both actin and tubulin had only a marginal impact on interfacial tension when compared to the protein-free condition (Figure 4b, black curve vs purple curve and gray curve vs green curve). Interestingly, while actin and lipids individually at the interface exhibited slow kinetics, their combined presence displayed an accelerated decrease (Figure 4b, yellow curve), suggesting a synergistic effect beyond mere additivity. We found this effect could not be countered via electrostatic or steric repulsion (i.e., the presence of charged or PEGylated lipids, respectively, Figure S4). These results imply that actin could, in line with previous research,^51^ quickly cover the surface of the droplets traversing the LOD, potentially impeding lipid monolayer formation and/or monolayer zipping. However, the full extent of this synergistic effect has yet to be uncovered. Furthermore, these results underscore the importance of the compositions of both inner solution and LOD as both affect mono- and bilayer formation.

### GUV Formation at the Oil–Water Interface Seems Size-Selective

Droplet formation in cDICE occurs on extremely short time scales; for the default conditions (i.e., 1900 rpm, 25 μL min^–1^), we observed droplets of approximately ∼70 μm in diameter being sheared off at a frequency of ∼2500 Hz. Theoretically, given a total encapsulation volume of 100 μL, >500,000 droplets are formed during a single experiment. Interestingly, this number does not correspond to the final number of GUVs produced using cDICE, as reported in other publications (∼1000 GUVs^31^). Furthermore, if these droplets larger than the finally observed GUVs (i.e., non-satellite droplets, ∼70 μm for the default conditions) do not subsequently shear to form smaller droplets as discussed above, these two observations together indicate a suboptimal GUV formation process downstream, whereby most droplets do not convert into GUVs at the oil–water interface and potential additional hidden mechanisms generating smaller droplets.

**Figure 5:**
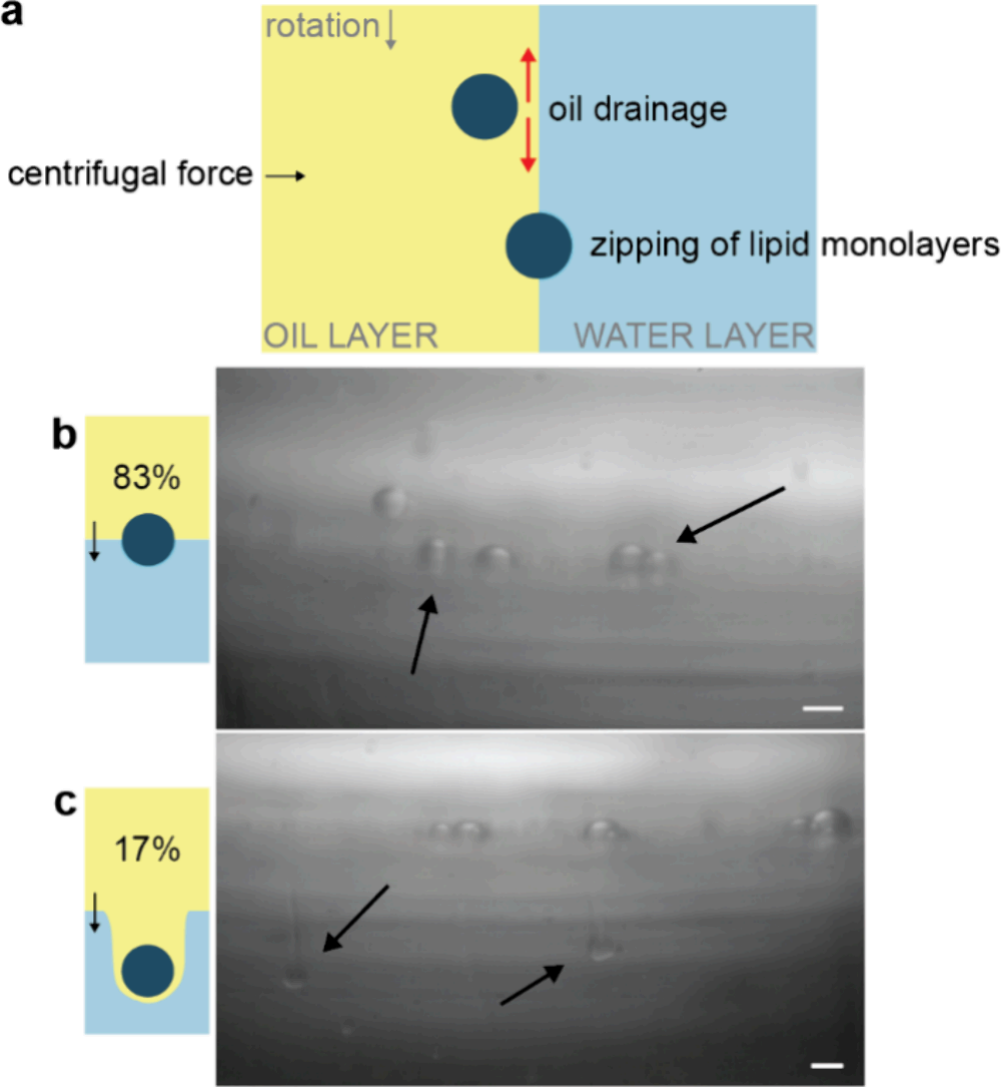
Droplet transfer through the oil–water interface is suboptimal (single column). (a) The LOD in between the approaching lipid monolayer-covered droplets and the oil–water interface needs to be drained for the two monolayers to zip together and for successful GUV formation to occur. (b) When droplets are not fully covered by a lipid monolayer when reaching the oil–water interface, successful transfer cannot occur and, instead, stationary, semitransferred droplets are observed (83.3%, *n* = 289). Scale bar indicates 100 μm. (c) When drainage of the oil layer between the approaching droplet and oil–water interface is insufficient, the formation of comet tails can be observed (16.7%, *n* = 58): a droplet distorts the oil–water interface and drags the LOD into the outer aqueous solution, hindering successful GUV formation. The scale bar is 100 μm. Droplets (*n* = 347) interacting with the oil–water interface are counted every 5th frame for 50 frames.

To look more closely at droplet-to-GUV conversion into GUVs in cDICE, we imaged the oil–water interface where the final step of GUV formation in cDICE occurs: droplets transfer through the oil–water interface, and two monolayers fuse together to form a bilayer (Figure 5a). As postulated by Abkarian et al.,^30^ the two monolayers can also form a pore, thereby causing the droplet to burst, resulting in no GUV being formed. We note that when we collected GUVs in cDICE experiments we observed that the outer solution after GUV generation also contained components of the inner solution, in agreement with the suggestion that a fraction of droplets burst at the oil–water interface.

In our experiments, we unfortunately did not observe a clear transfer of droplets through the interface or bursting of droplets, possibly because resolving GUV-sized droplets at the interface was not feasible with the limited imaging contrast of standard bright-field illumination. Instead, we made two other striking observations. First, we observed droplets several orders of magnitude larger than the typical size of GUVs, which were stationary on the oil–water interface (Figure 5b, Movies 8 and 9). These stationary droplets showed a decreased contrast on the side of the outer aqueous phase, suggesting a partial transfer across the interface. Since the transfer time of a droplet to the oil–water interface is inversely proportional to the radius of the droplets squared,^30^ the flight time of these larger droplets may be too short for lipids to fully adsorb on the interface. Consequently, no zipping mechanism is possible, leading to these larger droplets crowding the interface, as we observed in our video recordings.

A second observation was the formation of comet tails (Figure 5c): every one in six droplets (17%) coming at the oil–water interface passes the interface, dragging a tail of the oil solution into the outer aqueous solution, likely because the oil did not drain quickly enough and thus prevented monolayer fusion. Due to the difference in contrast with the outer aqueous phase, we infer that oil is still present between the part of the interface dragged into the outer phase and the droplet. The Bond number , where *a* is the acceleration and *r* is the radius of the droplet, represents the ratio of centrifugal force to surface tension force. For these large droplets, *Bo* is on the order of 1, meaning they will deform the interface, as observed in our video recordings, and drag the oil phase into the outer aqueous phase. This results in the observed comet tail formation and no GUV formation from the droplets undergoing this process.

As we find that the addition of protein to our inner solution significantly alters the characteristics of the solution and affects droplet formation at the capillary orifice, we asked how the increased viscosity and altered lipid adsorption kinetics might impact the transfer of droplets through the oil–water interface. The accelerated lipid adsorption due to the addition of protein does not lead to a decreased flight time of the droplets, but this mixed layer of lipids and proteins is not suitable for droplet transfer or monolayer zipping. On the other hand, the increased viscosity of the inner solution could influence the time scale of the drainage of the lubrication film, i.e., the LOD in between the droplet and the oil–water interface, required for successful monolayer zipping. Furthermore, the increased viscosity could reduce the flow caused by Marangoni stresses, which play a role in facilitating the zipping process.^30^

An approximate breakthrough condition for spherical objects of radius *a* to pass through an interface of interfacial tension γ is (ρ_*i*_–ρ_o_)Ω^2^*R*_o_*a*^2^/γ ≥ 3/2, where *R*_o_ is the distance between the axis of rotation and the location of the oil–outer solution interface.^55^ For small droplets of radius *a* ∼ 5 μm to cross the interface, a low surface tension on the order of γ ∼ 10^–6^ N m^–1^ is required. Such low surface tension has been reported for lipid bilayers,^56^ and therefore, if such small droplets are present in the oil phase, they can cross the interface to form GUVs. It should be emphasized that the breakthrough condition sets a criterion for the smallest droplet that can cross the interface. Any droplet larger than 10 μm in diameter would be expected to cross the interface as well and form larger GUVs. The fact that we do not observe GUVs of diameters larger than ∼20 μm,^31^ but do observe large droplets at the oil–water interface, suggests that the upper size limit for GUV formation might be controlled by membrane zipping and/or lipid coverage of the droplet/interface. Insufficient lipid coverage could, for example, lead to droplet/GUV shrinkage during GUV formation until the lipid density to form a bilayer is reached, thereby resulting in smaller GUVs than originally produced droplets.

We also compared cDICE GUV size distributions to those obtained by eDICE. eDICE is a recent adaptation of cDICE, the droplets are generated by vortexing, pipetting, or scraping, instead of using a capillary, but transferred through a second interface in a rotational chamber, identical to cDICE. Interestingly, we noticed that the final GUV size distributions were similar for the two methods,^57^ despite vastly different droplet size distributions being used as a starting point (Figure S5). Furthermore, we found GUV sizes to be remarkably similar for different membrane compositions in eDICE (Figure S6). Taken together, these cDICE and eDICE results indicate a yet unknown mechanism for size-selective droplet and/or GUV formation at the oil–water interface that promotes the production of similarly sized GUVs for a wide distribution of droplet sizes. For example, it is possible that GUVs form at the oil–water interface in cDICE and eDICE by pinching off larger droplets sitting at the interface. While we did not observe any event like this, we would expect this process to happen on a length scale (and possible time scale) beyond the resolution of our imaging setup.

## Conclusions

In summary, by designing and building a custom imaging setup to visualize droplet formation and droplet interface transfer in cDICE in real time, we were able to, for the first time, collect direct in situ imaging data to further understand the underlying mechanisms governing GUV formation in this technique. We found that droplet formation at the capillary orifice produced droplets that are much larger than the size of the final GUVs. For a capillary diameter of 100 μm, the formation of droplets in cDICE bears some similarities to the formation of droplets at T-junctions in microfluidics, a well-studied phenomenon.^58,59^ In such microfluidic channels, the geometric confinement provided by the channels leads to flow restrictions on the continuous phase at the origin of the squeezing pressure. This pressure promotes droplet breakup at much smaller values of *Ca* as compared to our experiments. However, there are similarities in the droplet formation regimes. For example, a decrease in droplet volume for increasing values of *Ca* has been widely reported.^58,59^ These studies have also reported a transition from a breakup droplet formation mechanism for low values of *Ca* to a dripping mechanism at higher *Ca*, whereby a long liquid filament of the dispersed phase forms and droplets pinch off at the end of the filament. This is in contrast to the use of smaller capillary openings in the original cDICE implementation, in the range of 2–25 μm,^30^ where the smaller inner diameter of the capillary leads to smaller droplet sizes by a combination of a smaller total interfacial force resisting the breakup of the droplet and a smaller Reynolds number. Only as a side process, smaller satellite droplets are being formed. Furthermore, we showed that the addition of protein to the inner solution increases its viscosity and changes interfacial tension dynamics, impacting droplet formation and likely also droplet interface transfer. Imaging of the oil–water interface revealed that droplet transfer is frequently stalled, large droplets remain stuck at the interface, and transfer exhibits a size-selectivity. This size-selectivity of droplet transfer to GUVs was further confirmed using eDICE, a variant of cDICE where no capillary is used, which yielded a similar size distribution despite vastly different droplets as input. We think therefore that, in addition to small (satellite) droplets being able to cross the interface to form GUVs, GUVs could also be produced by pinching off from the larger droplets we observed sitting at the rotating oil–water interface. While we did not directly observe this route of GUV formation, we also would not expect that we would be able to resolve GUVs leaving from the interface with our imaging setup. Further studies are needed to further elucidate the effect of lipid composition, including cholesterol or charged lipids, and different proteins or protein mixes. We believe the presented results can be of interest not only to cDICE but to other emulsion-based GUV formation methods as well as they suggest that GUVs do not just form as a simple conversion from droplets to GUVs at a second interface. Our study furthermore emphasizes the need for interdisciplinary collaboration to fully grasp the intricacies of the processes involved in emulsion-based GUV production methods to develop even more reliable and efficient methods for GUV production. We hope this research will serve as a stepping stone for future research, ultimately improving emulsion-based GUV formation.

## Methods

### Design and Fabrication of the Spinning Device

The cDICE device was identical to that by Van de Cauter et al.^31^ An additional opening underneath the spinning chamber was created by removing a part of the motor housing. This way, the light source could be placed directly below the spinning chamber to achieve transillumination. The design for the adjusted cDICE device is available on GitHub (https://github.com/GanzingerLab/cDICE_microscope).

### Fabrication of Spinning Chambers

Transparent, cylindrical chambers, 35 mm in diameter and 10 mm in height, were made from two lids of Petri dishes (Falcon REF 351008). To create a waterproof, closed chamber, the sides of the two lids were first sanded using sandpaper to create a rough surface, after which they were glued together using a thin layer of optical glue (Norland Optical Adhesive 81). After curing of the glue using UV light, the side of the chamber was wrapped with a strip of Parafilm. The chambers include a circular opening, 15 mm in diameter, at the top to allow facile access to the solutions with the capillary.

### General cDICE Experimental Workflow

While it is possible, and needed, to tweak various operational parameters to encapsulate a particular (non)biological system in cDICE, we chose to use the parameters established in a recent optimization study by Van de Cauter et al.^31^ as default conditions for cDICE. Specifically, we used a 100 μm diameter capillary, a rotation speed of 1900 rpm, and a flow rate through the capillary of 25 μL min^–1^. For the LOD, 18:1 1,2-dioleoyl-*sn*-glycero-3-phophocholine lipids were dispersed using chloroform in a 4:1 ratio of silicon oil:mineral oil (silicon oil—viscosity 5 cst (25 °C), Sigma-Aldrich; mineral oil—BioReagent, Sigma-Aldrich). A fused silica capillary tubing with a polyimide coating (TSP-100375, Molex LLC) was used to inject inner aqueous solutions. The general cDICE experimental workflow and preparation of LOD were based on Van de Cauter et al.^31^ The following parameters differed. The volume of the outer solution was increased to 1.07 mL to account for the difference in dimensions between the 3D printed spinning chambers, as used in Van de Cauter et al.,^31^ and the Petri dish spinning chambers that were used for imaging experiments, as mentioned above. Room humidity was not controlled during imaging experiments, and the chambers were spun for the entirety of the imaging experiments instead of a predetermined time. G-buffer (5 mM tris(hydroxymethyl)aminomethane hydrochloride (Tris–HCl) pH 7.8 and 0.1 mM calcium chloride (CaCl_2_), 0.02 mM adenosine triphosphate and 4 mM dithiothreitol) with 18.5% v/v OptiPrep was encapsulated in every experiment (to achieve a density difference between the inner and outer aqueous solutions), unless specified otherwise. For experiments with silanized capillaries, the tip of the capillary was submerged for 1 min in dichlorodimethylsilane (40140, Sigma-Aldrich), before removing the excess with nitrogen gas.

### Home-Built Imaging Setup

The light of a single LED (Luxeon V2, 315 lm@700 mA; used without lens) or a Lumencor light engine (SOLA 6-LCR-SB) was collected by a 200 mm focal length achromatic lens (Thorlabs AC254-200-A-ML; lens mount: Thorlabs CXY1). The setup was equipped with a 4× or 10× objective (Nikon Plan Fluor 4×/0.13 PhL DL and Nikon Plan Fluor 10×/0.30 ∞/0.17 WD 16, respectively) that was mounted on a Z-stage (Thorlabs CT1; adapter: Thorlabs SM1A10). X/Y motion control was provided by two translational stages with a step size of 25 mm (Thorlabs PT1). Images were recorded using a high-speed camera (Kron Technologies Chronos 2.1-HD and Photron FASTCAM SA4) that was mounted on the setup using a custom-designed 3D printed construction. The full setup was mounted on a Thorlabs cage system that was mounted on a breadboard (Thorlabs MB1030/M) to easily move the full setup over the cDICE device. The full component list and design plans, including an interactive 3D model of the setup, can be found on GitHub (https://github.com/GanzingerLab/cDICE_microscope).

### Droplet Size Analysis

Droplet size analysis was performed manually using the Fiji software.^60^ The image pixel size was derived from three independent measurements of the capillary opening, accounting for the capillary size uncertainty. Triplicate measurements were performed for a subset of each data set to quantify the measurement error. For each droplet, we then measured both the area and diameter, yielding two independent measurements of the droplet diameter, with the associated error calculated through error propagation. The large pixel size ((2.431 ± 0.105) μm) in comparison to the droplet size characterized, in combination with a measurement error of 2 μm, calculated from measuring a subset of data in triplicate, posed a limit on our analysis of smaller droplets. Additionally, the high speed of the process, resulting in motion blur and droplets quickly moving out of focus, as well as the limited contrast caused by the small difference in refractive index between the droplets and the surrounding medium (1.333 for water vs 1.403 for silicone oil), makes it difficult to distinguish the droplets from the background in the video recordings. Data visualization was achieved by Python-generated plots. The frequency was estimated using the mean droplet size and the flow rate of the inner solution. Note that for the analysis of droplet size and frequency we used video recordings in which the fluid tail did not adhere to the capillary surface (one experiment per condition).

### Viscosity Measurements

The dynamic viscosities of the solutions were measured on a Kinexus Malvern Pro rheometer. A stainless steel plate–plate geometry with 40 and 20 mm radii was used for buffer solutions and protein-containing solutions, respectively. Viscosity was measured every 5 s as a function of shear rate with a 2 min logarithmic viscometry ramp from 0.5 to 100 s^–1^. As expected for a simple viscous liquid, viscosities for a higher shear rate were constant. The values at 100 s^–1^ were used to calculate the reported viscosity of each solution. MRB80 buffers consist of 80 mM piperazine-N,N′-bis(2-ethanesulfonic acid) pH 6.8, 4 mM magnesium chloride (MgCl_2_), and 1 mM ethylene glycol-bis(β-aminoethyl ether)-N,N,N′,N′-tetraacetic acid.

### tensiometry Measurements

The pendant drop measurements were performed using a DSA 30S drop shape analyzer (Kruss, Germany) and analyzed with the Kruss Advanced software. Experimental conditions for G-buffer and actin-containing solutions were as described in Van de Cauter et al.,^31^ while changes for MRB80 buffer and tubulin-containing solutions are described below. Initially, a 2 μL droplet of aqueous solution is drawn in a LOD containing glass cuvette (Hellma Analytics), and then the volume of the droplet is adjusted to 8 μL using an automated dosing system from a hanging glass syringe with a needle diameter of 0.313 mm (Hamilton). As soon as the droplet reached its final volume, the droplet was analyzed (for 300 s at 25 fps for solutions containing tubulin and lipids and at 5 fps for the rest of the solutions) by automatic contour detection and fitted with the Young–Laplace equation to yield the interfacial tension. The densities of lipid oil solution (0.8685 mg/mL), G-buffer with 18.5% v/v OptiPrep (1.0574 mg/mL), and MRB80 with 1.75% w/v sucrose (1.0066 mg/mL) were used in the interfacial tension calculations. These densities were measured by weighing 1 mL of solution. For the G-buffer with OptiPrep, the density was estimated using the volume-weighted mean. The surface tension values were smoothed with a rolling mean of 1 s. Room humidity was not controlled. In several experiments, interfacial tension decreased very rapidly (abnormally), causing the droplets to detach as soon as they were formed. These measurements were discarded from the analysis.

## Supporting Information Available

Data of GUV yield and size distribution from cDICE and eDICE (PDF)
Droplet formation at the capillary orifice at a rotation speed of 1900 rpm (AVI)
Droplet formation at the capillary orifice at a rotation speed of 2700 rpm (AVI)
Droplet formation at the capillary orifice at a rotation speed of 1000 rpm (AVI)
Droplet formation at the capillary orifice for G-buffer (AVI)
Droplet formation at the capillary orifice for actin in G-buffer (AVI)
Droplet formation at the capillary orifice for MRB80 buffer (AVI)
Droplet formation at the capillary orifice for tubulin in MRB80 buffer (AVI)
Oil–water interface (AVI)
Droplet at oil–water interface (AVI)

## Supplementary Material

ao4c04825_si_001.pdf

ao4c04825_si_002.avi

ao4c04825_si_003.avi

ao4c04825_si_004.avi

ao4c04825_si_005.avi

ao4c04825_si_006.avi

ao4c04825_si_007.avi

ao4c04825_si_008.avi

ao4c04825_si_009.avi

ao4c04825_si_010.avi
